# Dilation of tricuspid valve annulus immediately after rupture of chordae tendineae in ex-vivo porcine hearts

**DOI:** 10.1371/journal.pone.0206744

**Published:** 2018-11-08

**Authors:** Keyvan Amini Khoiy, Kourosh T. Asgarian, Francis Loth, Rouzbeh Amini

**Affiliations:** 1 Department of Biomedical Engineering, The University of Akron, Akron, OH, United States of America; 2 Mid Atlantic Surgical Associates, Neptune, NJ, United States of America; 3 Department of Mechanical Engineering, The University of Akron, Akron, OH, United States of America; Texas A&M University System, UNITED STATES

## Abstract

**Purpose:**

Chordae rupture is one of the main lesions observed in traumatic heart events that might lead to severe tricuspid valve (TV) regurgitation. TV regurgitation following chordae rupture is often well tolerated with few or no symptoms for most patients. However, early repair of the TV is of great importance, as it might prevent further exacerbation of the regurgitation due to remodeling responses. To understand how TV regurgitation develops following this acute event, we investigated the changes on TV geometry, mechanics, and function of ex-vivo porcine hearts following chordae rupture.

**Methods:**

Sonomicrometry techniques were employed in an ex-vivo heart apparatus to identify how the annulus geometry alters throughout the cardiac cycle after chordae rupture, leading to the development of TV regurgitation.

**Results:**

We observed that the TV annulus significantly dilated (~9% in area) immediately after chordae rupture. The annulus area and circumference ranged from 11.4 ± 2.8 to 13.3 ± 2.9 cm^2^ and from 12.5 ± 1.5 to 13.5 ± 1.3 cm, respectively, during the cardiac cycle for the intact heart. After chordae rupture, the annulus area and circumference were larger and ranged from 12.3 ± 3.0 to 14.4 ± 2.9 cm^2^ and from 13.0 ± 1.5 to 14.0 ± 1.2 cm, respectively.

**Conclusions:**

In our ex-vivo study, we showed for the first time that the TV annulus dilates immediately after chordae rupture. Consequently, secondary TV regurgitation may be developed because of such changes in the annulus geometry. In addition, the TV leaflet and the right ventricle myocardium are subjected to a different mechanical environment, potentially causing further negative remodeling responses and exacerbating the detrimental outcomes of chordae rupture.

## Introduction

The tricuspid valve (TV) guides the blood from the right atrium to the right ventricle and prevents backflow during ventricular contraction. The leaflets and annulus of the TV undergo complicated dynamic deformations during normal cardiac cycles [1, 2]. Any disturbance in the normal deformation of the valve leaflets and/or annulus could lead to valvular regurgitation [3, 4] and changes in the valve’s mechanical response [5, 6]. In most cases, TV regurgitation—whether it is caused by primary valvular lesions (e.g., due to congenital malformations [7], trauma [8, 9] or degenerative diseases such as Marfan syndrome [10, 11]) or takes place as a result of other cardiovascular diseases (e.g. secondary to pulmonary hypertension [12])—will require surgical intervention [13].

While TV regurgitation is most often caused by chronic diseases, acute cases could occur following traumatic events such as vehicular accidents [14, 15]. In the majority of trauma-related cases, chordae rupture is the main lesion present [16]. Many accident victims also suffer from other injuries such as lacerations, fractures, and closed head injuries [17]. With these more severe concurrent injuries present immediately after trauma, the acute TV regurgitation can be easily overlooked when making a diagnosis. Although advances in diagnostic procedures such as echocardiography have improved in recent years [18], isolated TV regurgitation is often well tolerated, and most patients experience few or no symptoms in the weeks and months following the trauma [19, 20]. In fact, the average time from the trauma to the initial diagnosis of TV regurgitation is three years [21] (with the time to diagnosis ranging from within 15 days of the trauma to as long as 25 years later). However, early repair of the regurgitative TV following chordae rupture is of critical importance. Among many benefits, early repair may prevent further detrimental complications such as thickening and fibrosis of TV leaflets and/or changes in the sinus rhythm due to right atrial dilation [21].

Previous studies have shown that acute biomechanical changes in cardiac valves could induce remodeling responses that may negatively affect the valve structure, mechanical properties, and function [5, 22, 23]. Considering the importance of such acute events, in this study we aimed to identify how TV regurgitation develops immediately following chordae rupture. Since normal TV function relies on precise and complex interactions among the various components (i.e. annulus, leaflets, chordae), it is expected that chordae rupture disturbs the normal deformation of the valve annulus and leads to insufficient leaflet coaptation. It has been previously shown that valve insufficiency can lead to ventricular and annulus dilation due to remodeling responses. However, based on the assumption that intact chordae tendineae mechanically support/anchor the normal TV annulus, for the first time in this ex-vivo study we have shown that immediately following chordae rupture, the TV annulus dilates in porcine hearts.

## Methods

### Ex-vivo heart apparatus

**Fig 1:**
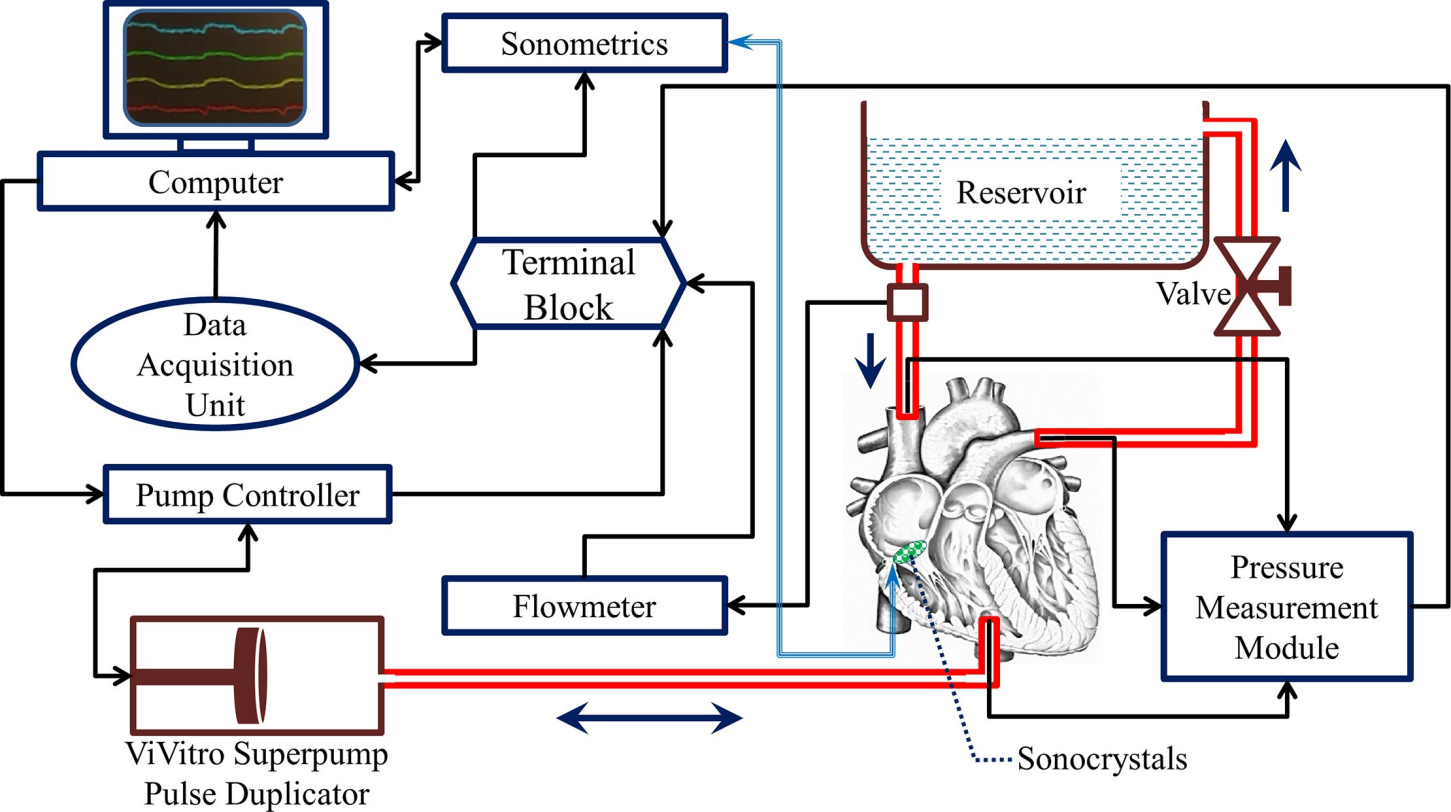
Schematic of the ex-vivo heart apparatus [24].

We have previously developed an ex-vivo passive beating heart apparatus to mimic TV deformation without the need to dissect the valve and remount the annulus [24]. The schematic of this apparatus is shown in Fig 1. In short, a positive displacement pump (SuperPump AR Series, Vivitro Labs, Inc., Victoria, BC, Canada) was utilized to induce passive beating in porcine hearts through pressure differences (without actively engaging the muscle tissue). The pump, heart, and a fluid reservoir filled with isotonic phosphate buffered saline (PBS) were connected together using tubes and tube fittings to build a closed hydraulic circuit, in which the pump could circulate the fluid from the reservoir into the heart and back to the reservoir. Backward movement of the pump piston (towards the left in Fig 1) causes the TV to open and pulls the PBS from the right atrium (which is connected to the reservoir) into the right ventricle. Forward movement of the pump piston (towards the right in Fig 1) causes the TV to close and the pulmonary artery valve to open by increasing the pressure inside the ventricle, pushing the PBS back into the reservoir through the pulmonary artery. As such, the pump is able to circulate the PBS throughout the system and generate movement and deformation of the TV leaflets and annulus. A transonic flowmeter (T108, Transonic Systems, Inc., Ithaca, NY) was used to monitor the flow rate, and three catheter-type pressure probes (SPR-524 probes with a PCU-2000 controller, ADInstruments, Colorado Springs, CO) were used to monitor the right atrial pressure, right ventricular pressure (RVP), and pulmonary artery pressure. Based on the requirements of the heart valve testing procedures established by the International Standard Organization (ISO 5840) and U.S. Food and Drug Administration guidelines, a standard waveform of the pump with frequency of 70 *bpm* was used during the experiment. The other parameters of the pump controller were set to ensure that the hydrodynamic pressure of the flow would closely match those of a heart under normal physiological conditions.

### Sample preparation

**Fig 2:**
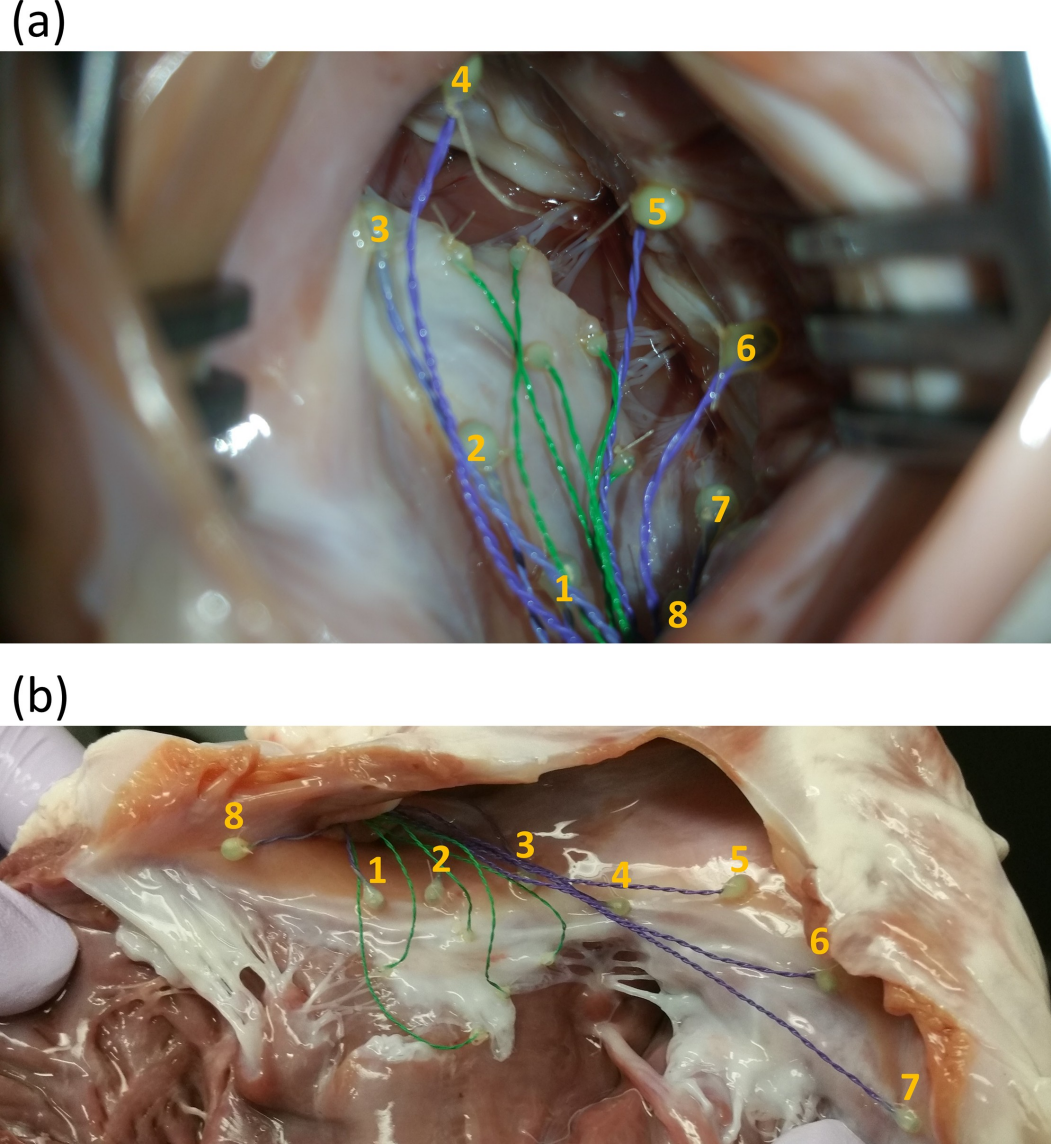
Eight sonocrystals (2 *mm* in diameter) sutured around the valve annulus (a) before the experiment and (b) after the experiment. The pulmonary side of the heart has been cut open for better visualization of the positions of the crystals.

Fresh porcine hearts were obtained from a local slaughterhouse (3-D Meats, Dalton, OH) and were transferred to the laboratory in bags filled with PBS that were covered with ice. Upon arrival at the lab, the hearts then were flushed out using PBS at room temperature and checked to ensure that no blood clots were stuck inside the chambers or in the area around the TV apparatus. In order to measure annulus deformation, a total of eight sonocrystals (Sonometrics Co., London, ON, Canada), 2 *mm* in diameter, were carefully sutured around the valve annulus (Fig 2). The suturing process was conducted through the superior vena cava, and the crystal wires were passed through the inferior vena cava to prevent any damage to the heart. To form a reference frame for calculating the positional data, three more sonocrystals, 3 *mm* in diameter, were attached to the outside of the myocardium close to the apex. A sonomicrometer (TRX Series 16, Somometrics Co., London, ON, Canada) was used to collect data from the sonocrystals. The pressure and flow signals were also collected via the sonomicrometer input channels to record all data in a synchronized manner. After setup of the system but prior to recording any data, an endoscopic camera (Snakescope SSVR-710) was sent into the right atrium through the superior vena cava to verify the accurate functionality of the valve apparatus. The positional data of the sonocrystals were recorded using a sampling rate of 100 *Hz* for a period of 20 seconds during each experiment. After recording the data for the intact TV in each experiment, the chordae tendineae of the septal leaflet towards the posteroseptal commissure were cut using surgical scissors, and the experiment was repeated to record the post chordae rupture (PCR) data. Eight hearts were tested under both intact and PCR conditions.

### Data analysis

**Fig 3:**
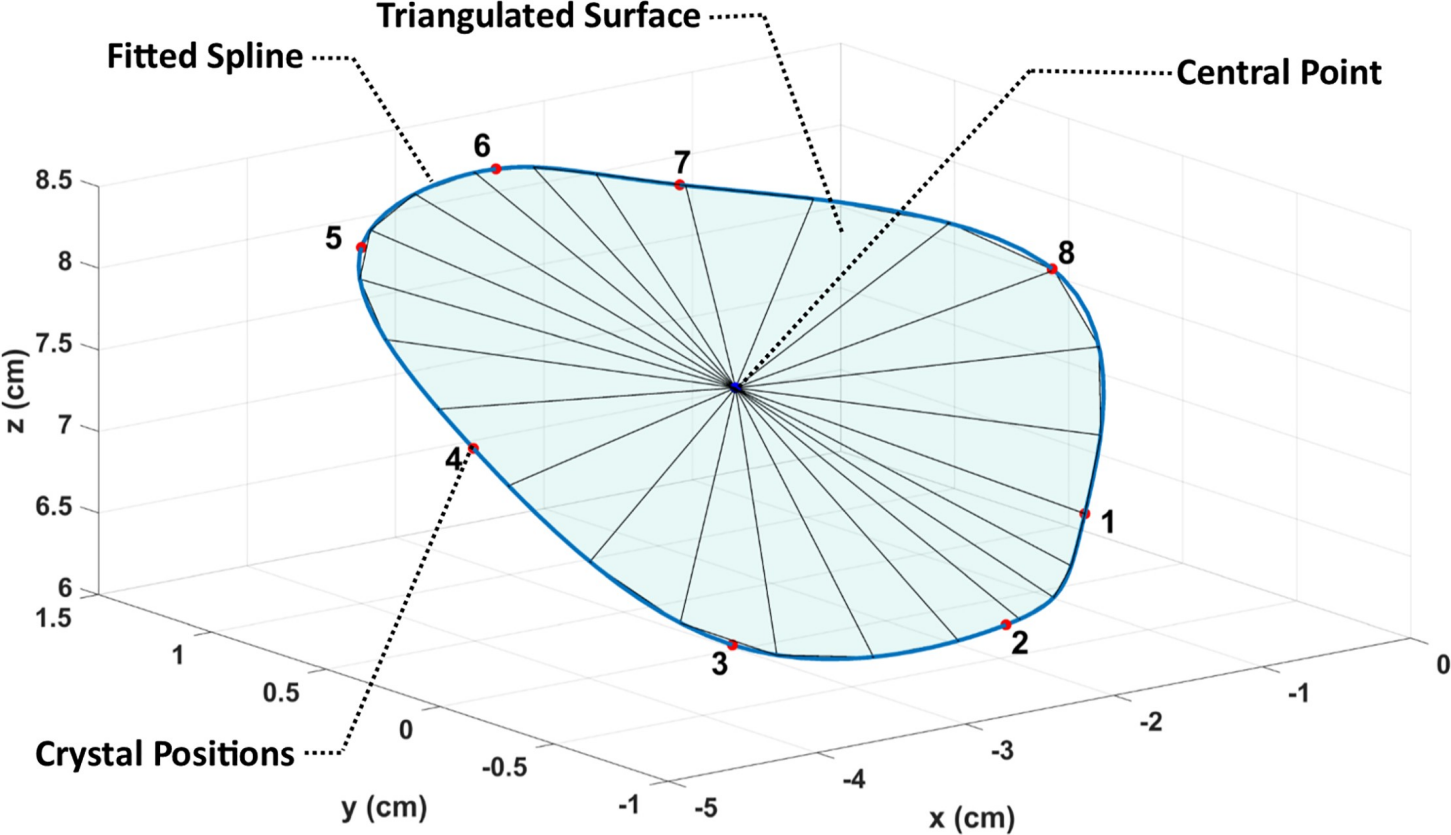
Method used to calculate the area, circumference, and radius of the annulus. A lower resolution of the original triangulation is presented for illustrative purposes.

In order to calculate the area and circumference of the annulus at each moment during the cardiac cycle, a cubic spline was fitted to the positional data [25, 26] of the crystals around the annulus (Fig 3). The length of the spline was calculated and considered as the circumference of the annulus at each moment [25]. The area of the annulus was approximated as follows:

1. The average of the three-dimensional position vectors collected by the sonocrystals was calculated as the center of the annulus.
2. A triangulated virtual surface was built by connecting 10,000 equally-spaced points on the spline representing the annulus to the calculated central point.
3. The sum of the surface areas of all the constructed triangles was calculated as an approximation for the area of the annulus.

The approximation procedure is illustrated in Fig 3 using a lower resolution of the points on a typical annulus for better illustrative purposes (24 points for illustrative purposes in Fig 3; the resolution used in the calculation was 10,001 points). The average of the measured distance between each point on the spline and the central point was calculated as the average radius of the annulus. The calculated area and circumference were also used to calculate the radius for comparison purposes by assuming that the annulus is a circle on a flat plane.

The following equation was used to calculate the dilation resulting from the chordae rupture:

$$\%D_{Q,t}=\frac{Q_{PCR,t}-Q_{I,t}}{Q_{I,t}}\times 100$$ (1)

where *Q* is the desired quantity; the subscripts *PCR* and *I* refer to PCR and intact conditions, respectively; subscript *t* indicates the time point in the cardiac cycle at which the calculation is being performed; and *D*_*Q*,*t*_ is the percentage change in dilation of the desired quantity *Q* at the specified time *t*.

To calculate the geometric changes during the cardiac cycle, the annulus area, circumference, and radius at the minimum RVP (minimum RVP was presumed to occur at the same location as the minimum right atrial pressure) of the intact condition were selected as the reference area (*A*_0_), reference circumference (*C*_0_), and reference radius (*R*_0_), respectively. Next, using Eq (2), we calculated the changes in area, circumference, and radius for the annulus under both intact and PCR conditions.

$$\%C_{Q}=\frac{Q_{t}-Q_{0}}{Q_{0}}\times 100$$ (2)

where *Q*_*t*_ is the desired quantity at the current time, *Q*_0_ is the initial (reference) value of this quantity, and *C*_*Q*_ shows the percentage change. For comparison purposes, we also calculated the approximate changes in annulus anterior segment (AAS), annulus posterior segment (APS), and annulus septal segment (ASS) using the position of the markers attached to each segment.

Finally, we developed an average annulus curve from the measured 3D data to evaluate its shape. As the data was recorded independently for each tricuspid valve annulus based on its own reference frame and coordinate system, registration of marker positional data from different annuli was necessary to match the corresponding marker positions with a minimum error for averaging. Therefore, using singular value decomposition, the measured positional data for all tricuspid valve annuli were transformed to closely register the corresponding marker points, and the resulting data points were averaged to develop an average annulus curve similar to the ones developed in previous studies [25].

### Statistical analysis

All data presented in this paper are reported in the form of mean ± standard deviation. Although much intervariability was observed among the measured values of different subjects (more details in this regard can be found in the Results section), the standard deviation due to measurement errors for 22 consecutive cardiac cycles was ~0.1% of the mean value of the measured quantity. Nevertheless, to minimize the intervariability due to measurement errors for each sample, the measured data of all 22 consecutive cardiac cycles were averaged at the corresponding time points in the cardiac cycle and were used for analysis. The Wilcoxon signed rank test was used for all statistical analysis, where any result with *p* < 0.05 is considered to be statistically significant.

## Results

### Pressure

**Fig 4:**
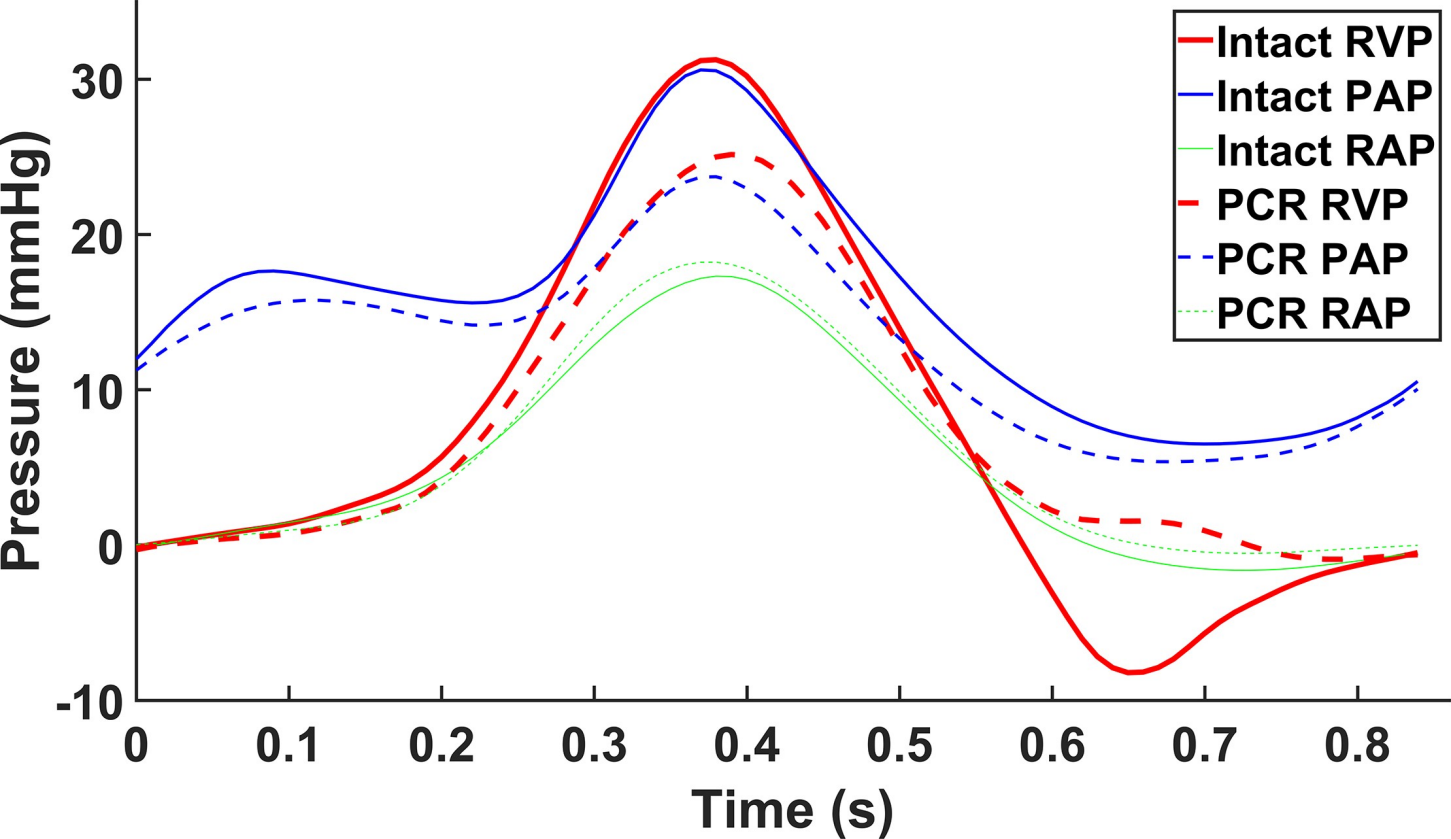
Average right ventricular pressure (RVP), pulmonary artery pressure (PAP), and right atrial pressure (RAP) measured for the intact and post chordae rupture (PCR) cases.

The average recorded RVP, pulmonary artery pressure (PAP), and right atrial pressure (RAP) are shown in Fig 4 for the intact and PCR cases. As illustrated in this figure, the average measured pressures in this ex-vivo setup closely match the in-vivo ones [27]. The range of pressure values for the intact case were approximately from 0 to 30 *mm Hg* and from 6 to 30 *mm Hg* for RVP and PAP, respectively. However, the pressures for RVP and PAP after chordae rupture ranged from 0 to 25 *mm Hg* and from 5 to 24 *mm Hg*, respectively. The range for the RVP closely matched the range reported for the porcine RVP in the literature [28–31]. We were not able to find reported PAP or RAP values for porcine hearts; however, the recorded pressure values closely matched those measured in human hearts [27].

### Annulus area, circumference, and radius values

**Fig 5:**
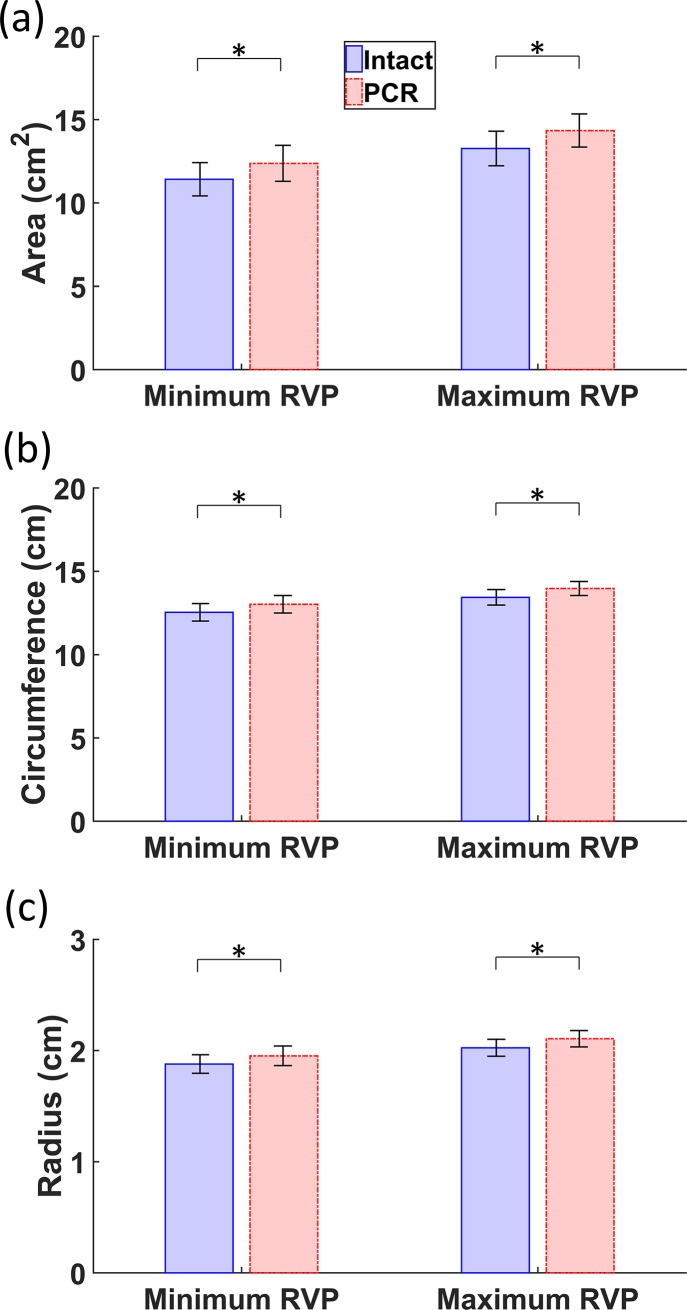
Comparison of the average values of (a) the area, (b) circumference, and (c) radius between the intact and post chordae rupture (PCR) conditions at minimum and maximum right ventricular pressure (RVP). The Wilcoxon signed rank test p-values for area, circumference, and radius were 0.01 at maximum RVP and 0.04 at minimum RAP. The asterisks (*) show significant differences (*p* < 0.05, Wilcoxon signed rank test). Error bars show the standard errors.

The results obtained for the area, circumference, and radius at the minimum and maximum RVP for intact and PCR ex-vivo porcine hearts are listed in Tables 1–3. Throughout the cardiac cycle, in intact hearts, the mean area, circumference, and radius of the annulus ranged from 11.4 ± 2.8 to 13.3 ± 2.9 *cm*^2^, from 12.5 ± 1.5 to 13.5 ± 1.3 *cm*, and from 1.9 ± 0.2 to 2.0 ± 0.2 *cm*, respectively. After chordae rupture, the mean area, circumference, and radius of the annulus ranged from 12.3 ± 3.0 to 14.4 ± 2.9 *cm*^2^, from 13.0 ± 1.5 to 14.0 ± 1.2 *cm*, and from 1.9 ± 0.2 to 2.1 ± 0.2 *cm*, respectively. The numbers provided here are the average of the minimum and maximum values of the quantities, while Tables 1 to 3 list the values at minimum and maximum RVP; thus, there might be a slight difference between the averages and standard deviations provided here and those presented in Tables 1 to 3. Significant increases in the annulus area, circumference, and radius were observed following chordae rupture (Fig 5; *p* = 0.01 for values measured at maximum RVP and *p* = 0.04 for values measured at minimum RAP, according to the Wilcoxon signed rank test). A segment-specific statistical analysis revealed that the APS did not vary significantly after chordae rupture (*p* = 0.38 for values measured at maximum RVP and *p* = 0.64 for those measured at minimum RAP, according to the Wilcoxon signed rank test), while the AAS and ASS increased significantly (*p* = 0.02 for AAS values measured at maximum RVP, *p* = 0.04 for AAS values measured at minimum RAP, and *p* = 0.02 for ASS values measured both at maximum RVP and at minimum RAP, according to the Wilcoxon signed rank test). Table 3 also lists the radii estimated from the calculated areas and circumferences (*R*_*A*_ and *R*_*C*_) when the annulus was considered as a flat circle. These estimated radii values, especially those calculated from the areas, were quite similar to those calculated using the previously explained method.

**Table 1:** Calculated area at minimum and maximum right ventricular pressure (RVP) for intact and post chordae rupture (PCR) conditions. The values are presented for all eight hearts used in the experiments along with the average (AVG) and standard deviation (STD). Comparing the average values showed an increase in the area post chordae rupture.

	Area (*cm*^2^)			
	At Minimum RVP			At Maximum RVP
Heart No.	Intact	PCR	Intact	PCR
**1**	12.7	12.7	13.7	14.0
**2**	8.8	8.7	11.5	12.1
**3**	8.8	9.9	10.9	12.2
**4**	13.8	13.9	15.3	15.5
**5**	8.0	9.4	9.8	11.5
**6**	16.2	17.7	19.1	20.2
**7**	11.6	14.7	12.8	15.5
**8**	11.5	12.0	13.0	13.6
**AVG**	11.4	12.4	13.3	14.3
**STD**	2.8	3.0	2.9	2.8

**Table 2:** Calculated circumference at minimum and maximum right ventricular pressure (RVP) for intact and post chordae rupture (PCR) conditions. The values are presented for all eight hearts used in the experiments along with the average (AVG) and standard deviation (STD). Comparing the average values showed an increase in the circumference post chordae rupture.

	Circumference (*cm*)			
	At Minimum RVP			At Maximum RVP
Heart No.	Intact	PCR	Intact	PCR
**1**	13.2	13.2	13.7	13.8
**2**	11.0	11.0	12.5	12.9
**3**	10.9	11.7	12.2	12.9
**4**	13.7	13.8	14.4	14.5
**5**	10.9	11.8	11.9	12.9
**6**	14.9	15.5	16.0	16.4
**7**	12.9	14.2	13.4	14.6
**8**	12.8	13.0	13.4	13.8
**AVG**	12.5	13.0	13.4	14.0
**STD**	1.5	1.5	1.3	1.2

**Table 3:** Calculated radius using the triangulation method (*R*) along with the radii calculated from the area (*R*_*A*_) and circumference (*R*_*C*_), using the assumption of flat annuli, at minimum and maximum right ventricular pressure (RVP) for intact and post chordae rupture (PCR) conditions. The values are presented for all eight experimental hearts along with the average (AVG) and standard deviation (STD). Comparison between *R*, *R*_*A*_, and *R*_*C*_ showed that the three different methods of calculating the radius produced the same results.

	Radius (*cm*)											
	At Minimum RVP						At Maximum RVP					
Heart	Intact			PCR			Intact			PCR		
No.	*R*	*R*_*A*_	*R*_*C*_	*R*	*R*_*A*_	*R*_*C*_	*R*	*R*_*A*_	*R*_*C*_	*R*	*R*_*A*_	*R*_*C*_
**1**	2.0	2.0	2.1	2.0	2.0	2.1	2.1	2.1	2.2	2.1	2.1	2.2
**2**	1.7	1.7	1.8	1.6	1.7	1.8	1.9	1.9	2.0	1.9	2.0	2.1
**3**	1.7	1.7	1.7	1.8	1.8	1.9	1.9	1.9	1.9	2.0	2.0	2.1
**4**	2.1	2.1	2.2	2.1	2.1	2.2	2.2	2.2	2.3	2.2	2.2	2.3
**5**	1.6	1.6	1.7	1.7	1.7	1.9	1.7	1.8	1.9	1.9	1.9	2.1
**6**	2.3	2.3	2.4	2.4	2.4	2.5	2.4	2.5	2.5	2.5	2.5	2.6
**7**	2.0	1.9	2.1	2.2	2.2	2.3	2.0	2.0	2.1	2.2	2.2	2.3
**8**	1.9	1.9	2.0	1.9	2.0	2.1	2.0	2.0	2.1	2.1	2.1	2.2
**AVG**	1.9	1.9	2.0	2.0	2.0	2.1	2.0	2.1	2.1	2.1	2.1	2.2
**STD**	0.2	0.2	0.3	0.3	0.2	0.2	0.2	0.2	0.2	0.2	0.2	0.2

### Annulus dilation due to the chordae rupture

**Fig 6:**
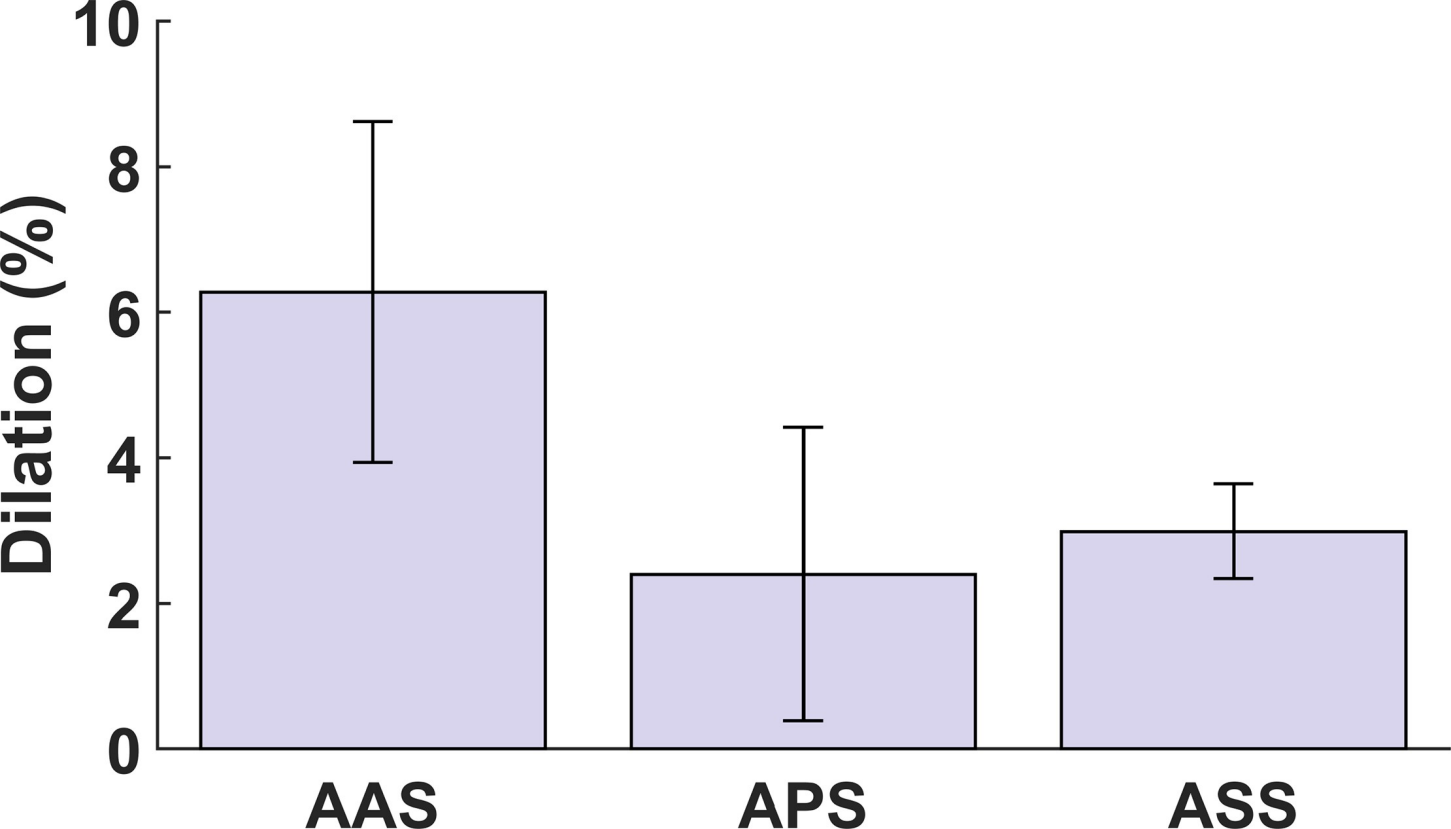
Comparison of the dilation (due to the chordae rupture) between annulus anterior segment (AAS), annulus posterior segment (APS), and annulus septal segment (ASS) at maximum right ventricular pressure (RVP). The Wilcoxon signed rank test p-values were 0.55, 0.38, and 0.74 between the AAS and APS, the AAS and ASS, and the APS and ASS, respectively. No significant differences were observed (*p* > 0.05, Wilcoxon signed rank test). Error bars show the standard errors.

Table 4 shows the increase in area, circumference, and radius of the annuli (i.e., a measure of annuli dilation) due to the chordae rupture averaged over all ex-vivo porcine hearts at maximum RVP. The area of the annuli dilated an average of 8.8% at maximum RVP. The dilation at this point for both the circumference and the radius was approximately 4% on average. The segment-specific dilations were also calculated and are presented in Table 5 for the AAS, APS, and ASS of the annuli. The largest average dilation in the circumference of the annuli (6.3%) occurred at the AAS, whereas the lowest average dilation (2.4%) occurred at the APS. Fig 6 shows a comparison among the average dilations (at maximum RVP) of three annulus segments. Statistical analyses showed that there was no significant difference between the dilations in the different segments of the annuli (Fig 6).

**Table 4:** Geometric dilation in area, circumference, and radius of the heart annuli due to chordae rupture at maximum right ventricular pressure (RVP) calculated using Eq (1) along with the average (AVG) and standard deviation (STD) for each quantity.

	Dilation at Maximum RVP (%)		
Heart No.	Area	Circumference	Radius
**1**	2.4	1.0	1.1
**2**	5.2	3.2	2.5
**3**	11.9	6.4	5.1
**4**	1.0	0.4	0.9
**5**	17.1	8.1	8.3
**6**	5.9	2.6	3.3
**7**	20.8	8.6	9.3
**8**	5.9	2.5	2.9
**AVG**	8.8	4.1	4.2
**STD**	7.1	3.2	3.2

**Table 5:** Dilation in the length of annulus anterior segment (AAS), annulus posterior segment (APS), and annulus septal segment (ASS) due to the chordae rupture at maximum right ventricular pressure (RVP) calculated using Eq (1) along with the average (AVG) and standard deviation (STD) for each quantity. The largest dilation occurred at the AAS.

	Dilation at Maximum RVP (%)		
Heart No.	AAS	APS	ASS
**1**	-1.2	2.3	1.9
**2**	7.6	-1.8	3.8
**3**	19.1	-5.2	-0.3
**4**	0.7	-1.4	5.6
**5**	10.2	7.6	4.5
**6**	2.8	1.4	3.8
**7**	9.2	12.8	2.3
**8**	1.8	3.5	2.4
**AVG**	6.3	2.4	3.0
**STD**	6.6	5.7	1.8

### Changes in annulus geometry throughout the cardiac cycle

**Fig 7:**
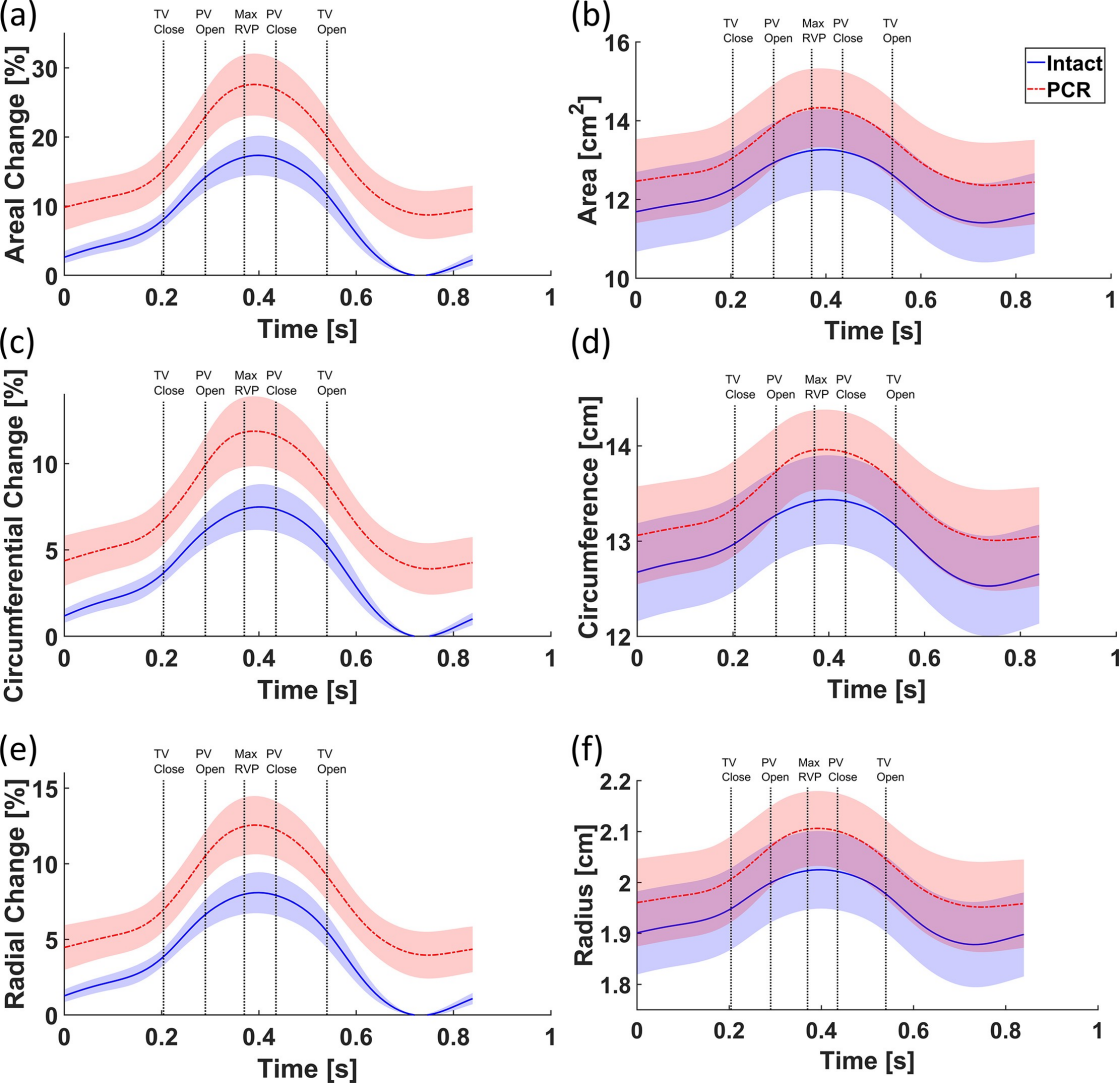
Changes in (a) area, (c) circumference, and (e) radius as well as the absolute values of (b) area, (d) circumference, and (f) radius throughout the cardiac cycle averaged over all the annuli for intact and post chordae rupture (PCR) conditions. The shaded regions show the standard errors. The temporal position of the maximum right ventricular pressure (RVP) as well as the opening and closure of the tricuspid and pulmonary valves for the intact case are shown in the graphs as a better illustration of the deformations that occur throughout the cardiac cycle.

**Fig 8:**
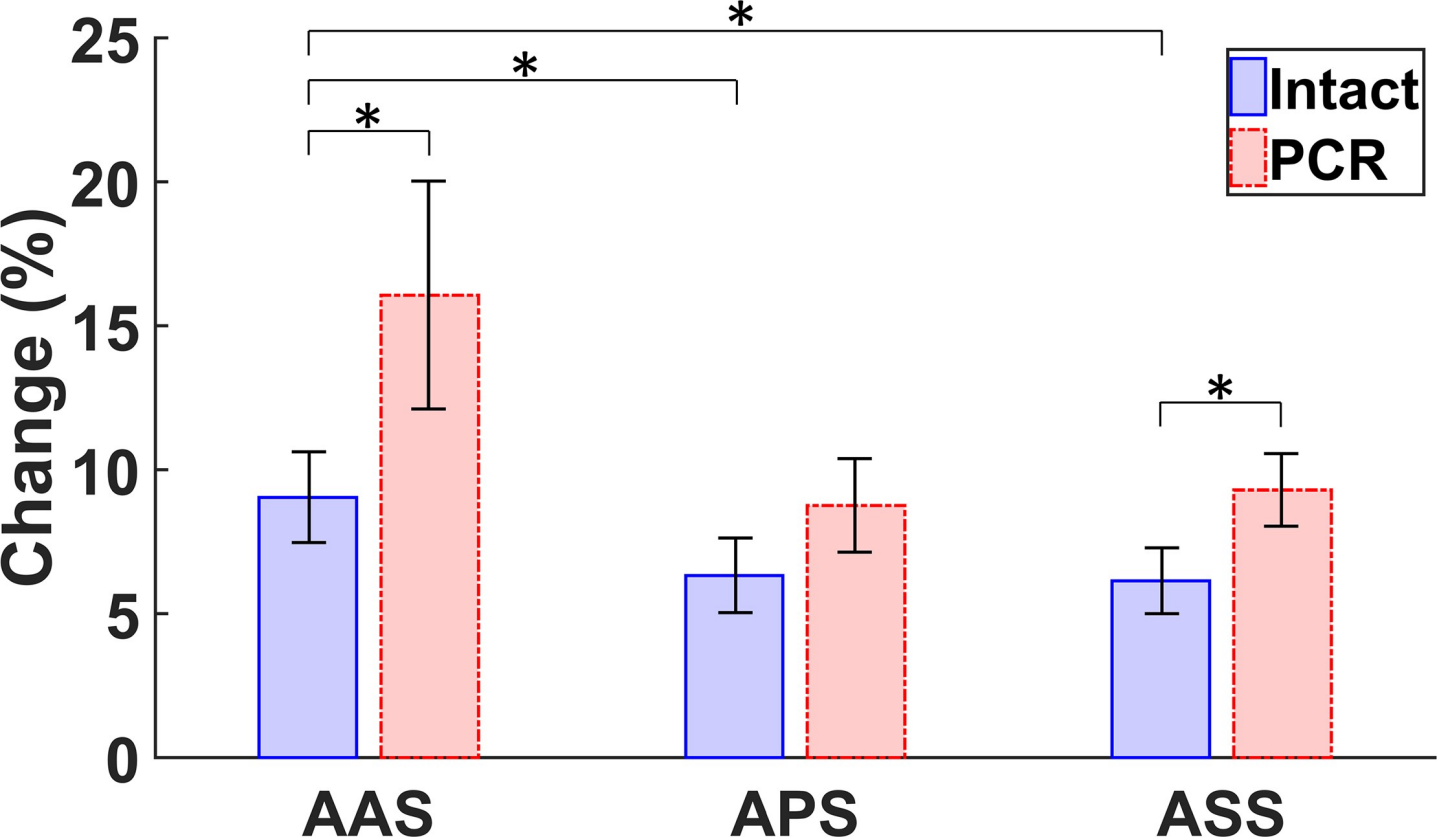
Comparison of the change in the length of the annulus anterior segment (AAS), annulus posterior segment (APS), and annulus septal segment (ASS) in intact and post chordae rupture (PCR) conditions at maximum right ventricular pressure (RVP). The PCR values include the dilation as well. For a comparison of the change in length between the intact and PCR conditions, the Wilcoxon signed rank test was used; p-values were 0.02 for AAS and ASS and 0.38 for APS. The p-values were 0.03, 0.02, and 0.84 for the comparison of the change in length for the intact case between the AAS and APS, the AAS and ASS, and the APS and ASS, respectively. The asterisks (*) indicate significant differences (*p* < 0.05, Wilcoxon signed rank test). Error bars show the standard errors.

The changes in metrics of each annulus throughout the cardiac cycle were calculated using Eq (2), and the resulting values were averaged over all eight annuli, as shown in Fig 7. Table 6 also shows the average changes (calculated using Eq (2)) at maximum RVP for normal and PCR ex-vivo porcine hearts. From the minimum to the maximum RVP, the annulus area increased by 17 and 18% in intact and PCR hearts, respectively. However, when accounting for the annulus dilation following chordae rupture, the maximum change in area was found to exceed 27%. In other words, the same PCR annuli showed more dilation if the changes were always referenced to the corresponding intact hearts. The maximum changes in circumference and radius (in both intact and PCR conditions) ranged roughly from 7.5% to 8.5%. Again, accounting for chordae rupture–induced annulus dilation, approximately 12% maximum circumferential and radial changes were calculated. The maximum change in segment-specific circumferences during the cardiac cycle are also shown in Table 6. From this table, it can be noticed that the AAS of the annulus circumference experienced a larger average change during the cardiac cycle when compared with APS and ASS. Fig 8 shows the change in the length of three segments of the annulus at maximum RVP for both intact and PCR conditions. For the intact hearts, the changes in the length of the AAS during the cardiac cycle were significantly higher than those for the other two segments (*p* = 0.03 for the comparison between AAS and APS, *p* = 0.02 for the comparison between AAS and ASS, and *p* = 0.84 for the comparison between APS and ASS, according to the Wilcoxon signed rank test). There was, however, no significant difference between changes in the lengths of different segments after chordae rupture (The smallest p-value was 0.08, according to the Wilcoxon signed rank test for each comparison).

**Table 6:** Average geometric changes at maximum right ventricular pressure (RVP) for intact and post chordae rupture (PCR) conditions calculated using Eq (2). The last column shows the percentage of the change in geometric parameters with intact-to-PCR dilation included in calculations. The geometrical parameters at minimum RVP were selected as the reference to calculate the changes.

	Geometrical Change at Maximum RVP (%)		
Quantity	Intact	PCR	PCR with Dilation
**Areal**	17.2	18.1	27.6
**Circumferential**	7.4	7.9	11.9
**Radial**	8.0	8.6	12.6
**Change in AAS**	9.0	9.6	16.1
**Change in APS**	6.3	6.9	8.8
**Change in ASS**	6.1	6.7	9.3

### Annulus curve

The resulting average tricuspid valve annulus has the shape of a nonplanar saddle curve. The maximum points of this curve were on the AAS and APS close to the anteroseptal and posteroseptal commissures, respectively. One of the minimum points, on the other hand, was approximately placed at the middle of the ASS, and the other was on the AAS close to the anteroposterior commissure. These observations are consistent with those reported for the in-vivo tricuspid valve annulus in an ovine model [1].

## Discussion

The positional data obtained from the sonocrystals sutured around the ex-vivo porcine heart annulus were used to analyze the annulus deformation during the cardiac cycle. The effects of the chordae rupture were also investigated on this deformation by cutting one of the septal chordae proximal to the posterior commissure. The analyses showed that if we consider the annulus as a flat circle and use the calculated area and circumference to estimate the radius, the estimated values are comparable to the values for the radius that were calculated directly from the annulus geometry.

Throughout the cardiac cycle, the geometry of the annulus alters considerably such that from the minimum RVP to the maximum RVP, the area roughly experiences a 20% increase, and the circumference extends approximately 8%. Our analysis showed that these deformations do not occur uniformly along the annulus. For example, the AAS experiences the largest deformation (about 9% on average). In most TV repair procedures, a prosthetic annuloplasty ring is used to decrease the annulus size and improve the valve hemodynamics [32]. Considering the dynamic deformation of the TV annulus observed in our study, further research is needed to identify how the valve deformation changes following ring annuloplasty [33].

Due to the inherent differences between the in-vivo and the ex-vivo cases, the results reported in this study might not exactly match the in-vivo outcomes. The absence of muscular contraction (including those of the myocardium and papillary muscles) and the lack of the interaction between the right and left ventricles in the ex-vivo experiments might affect the results in comparison to those of the in-vivo cases. As such, much caution should be taken in interpretation of our results. For example, it has been reported in in-vivo studies that the annulus dimensions decrease during the systole [2, 25]; for the ex-vivo case, however, they increase during the systole. In actively beating hearts, the contraction of the heart muscles decreases the annulus as well. In contrast, in passively beating hearts, no active contraction is present, and the increased ventricular pressure causes the annulus to expand during the systole. In particular, unlike our ex-vivo results, Rausch and colleagues recently observed 7.17 ± 1.93 *cm*^2^ as the minima of the annular area during systole in in-vivo ovine hearts, which was significantly smaller than the annular area during diastole (i.e., 8.65 ± 1.98 *cm*^2^) in the same hearts [25]. A similar trend was also observed for the measured perimeters in this in-vivo model (i.e., 10.2 ± 1.28 *cm* systolic value as compared to a significantly smaller diastolic value of 11.2 ± 1.27 *cm*). It is worth noting that quantitative comparison of the in-vivo active beating heart measurements [25] with our ex-vivo passive beating heart measurements should be conducted with caution due to the inherent differences in ovine versus porcine models.

In addition, in many different types of soft tissues, even in those that are not as mechanically active as cardiac muscles, the mechanical properties are different in the ex-vivo setups as compared to those measured in the native in-vivo environment [34]. Such differences have been attributed to the lack of perfusion and metabolic activities in the ex-vivo environments. As such, potential differences in the mechanical responses of the tissues in the in-vivo versus ex-vivo setups should also be considered in the interpretation of our results. Nevertheless, during the diastole, when the right ventricle is expected to be at its least active state, the measured ex-vivo area and circumference values (11.4 ± 2.8 *cm*^2^ and 12.5 ± 1.5 *cm*, respectively) closely matched those of the in-vivo measurements (8.65 ± 1.97 *cm*^2^ and 11.1 ± 1.27 *cm*, respectively) [25]. It should be noted that since plane projection of the three-dimensional geometry was used to calculate the area in the aforementioned in-vivo study, the area calculated in our study was slightly larger, as was expected. Furthermore, Fawzy et al. [2] reported that most changes in circumference occur in the anterior segment of the annulus in the in-vivo hearts, which is consistent with the findings of our study (Fig 8).

In atrioventricular valves, the structures of the chordae tendineae and papillary muscles anchor the valve leaflets and prevent them from billowing into the atrium during ventricular contraction [32]. As such, one would expect that regurgitation might occur when such constraints are removed from the leaflet(s) following chordae rupture. However, it is not just the billowing effects that are prevented by the parachute-like structure of the chordae tendineae and the papillary muscles. Our experiments showed that there exists a mechanical interdependency among TV chordae tendineae, leaflets, and annulus. We observed that immediately after chordae rupture, the dynamic deformation of the TV annulus changed extensively, with a significant increase in the annulus area, circumference and radius. Annulus dilation, whether it develops over time or occurs acutely (as in the case of trauma-induced chordae rupture), is expected to change the coaptation of the valve leaflets and induce regurgitation [13]. Our measurements showed that, on average, the flow into the right atrium decreased by 26% immediately after the septal chordae tendineae close to the posteroseptal commissure was cut. Moreover, the maximum RVP dropped from 30 mm Hg to 25 mm Hg, which is a 17% decrease. A similar decrease was observed in the PAP. These changes show how chordae rupture can alter the hemodynamics of the heart.

Recent *ex-vivo* studies have shown that the TV annulus and leaflets are under tension [24, 35]. An increase in the annulus area following chordae rupture may change the annulus tension and alter the homeostatic mechanical environment to which the leaflets and the myocardium surrounding the annulus are subjected. The homeostatic mechanical environment is extremely important for the normal function of the TV and its surrounding tissues and can alter its normal mechanical properties [5, 24]. In all types of cardiac valves, valve interstitial cells reside within the leaflet tissue [36–40]. The valve interstitial cells, by means of protein synthesis and enzymatic degradation, maintain the structural integrity of the leaflet tissue. In all types of soft tissues, collagen type I, which is the main load-bearing protein of the extracellular matrix, scales with tissue stiffness [41]. The in-vivo valve interstitial cells respond to changes in mechanical loading and alter the tissue stiffness via collagen synthesis or degradation. For example, in murine TVs, increasing the mechanical load to which the leaflets are subjected led to increased mRNA amounts of both collagen type I and III as well as to higher collagen turnover [42]. Conversely, in an ovine model, mitral valve collagen content was decreased when the ventricular pressure (and consequently the mechanical loading on the leaflets) decreased [22].

It is worth noting that cells are not the only components that are sensitive to changes in mechanical loading [43]. While large mechanical strains could increase the rate of extracellular matrix catabolism in cardiac valves [44], studies have shown that the extracellular matrix collagen is more stable and degrades more slowly under homeostatic mechanical strain [45–47]. Considering such an important influence of the tissue mechanical milieu on short-term as well as long-term valve responses, it is essential to not ignore dilation in the annulus following chordae rupture. Surely, further *in-vivo* animal studies are necessary to better examine the long-term effects of chordae rupture on TV function and structure. It is, however, expected that, in addition to the immediate effects of annulus dilation in generating secondary tricuspid regurgitation, in the long term, changes in the mechanical environment of the TV leaflet and right ventricle myocardium could cause further negative remodeling responses and exacerbate the detrimental outcomes of chordae rupture.

In summary, we employed an ex-vivo heart setup and measured the deformation of porcine TV annuli during simulated cardiac cycles. Regurgitation was induced by cutting the chordae tendineae of the septal leaflets. For the first time, we observed that the TV annulus dilates immediately after the rupture of the chordae tendineae. Although the TV may be initially asymptomatic, instantaneous annulus dilation following chordae rupture could lead to exacerbation of the TV regurgitation and potentially to mechanically-induced remodeling responses in the TV leaflets, the remaining intact chordae tendineae, the papillary muscles, and/or the ventricular myocardium. More careful examinations and early surgical interventions might be necessary to prevent mid-term/long-term negative effects of mechanically-induced remodeling in asymptomatic TVs following the rupture of the chordae tendineae.
